# Particulate matter on two *Prunus* spp. decreases survival and performance of the folivorous beetle *Gonioctena quinquepunctata*

**DOI:** 10.1007/s11356-018-1842-4

**Published:** 2018-03-30

**Authors:** Adrian Łukowski, Robert Popek, Radosław Jagiełło, Ewa Mąderek, Piotr Karolewski

**Affiliations:** 1 Institute of Dendrology Polish Academy of Sciences, Parkowa 5, 62-035 Kórnik, Poland; 2 Faculty of Forestry, Poznań University of Life Sciences, Wojska Polskiego 71c, 60-625 Poznań, Poland

**Keywords:** Folivorous insect, Insect performance, Leaf beetle, PM accumulation, *Prunus padus*, *Prunus serotina*

## Abstract

Woody plants growing along streets and construction sites play an important role in removing harmful particulate matter (PM). Researchers rarely consider the impact of different types and size fractions of PM deposited on the leaves on insect folivores. We determined differences in the accumulation of cement and roadside PM on the leaves of two *Prunus* species (*P. padus* and *P. serotina*) with different leaf surface structures. We also determined the effect of PM on the beetle *Gonioctena quinquepunctata*, the main pest of these plants. Saplings were artificially dusted in greenhouses and leaves were utilised for larval and adult insect stages feeding in laboratory conditions. Road PM accumulated in greater amounts than did cement PM, regardless of plant species. For both PM sources, *P. padus* accumulated twofold more than did *P. serotina*. Insect survival was negatively affected by PM pollution; however, neither *Prunus* species nor PM source variant significantly affected masses of larvae and pupae, duration of larval and pupal development or relative growth rates. The experiment showed strong negative influences of PM were noted only for adult insects, due to the grazing period being longer than that in larvae. The mass of adult insects and the efficiency of conversion of ingested food (ECI) were lower for insects exposed to PM than those for control insects. Insects compensated for lower ECI by eating a greater total amount of food (TFE). Adult insects gained significantly higher mass when fed with *P. serotina* than with *P. padus*. The effect of PM on analysed plant metabolites was insignificant. Only *Prunus* sp. and date of collection affected the level of condensed tannins and total phenols. Our results indicate that, when investigating the effect of the host plant on folivore performance, the accumulation of PM, as well as its type and quantity, should be taken into account.

## Introduction

Air pollution is an important global problem, having a crucial impact on the health and life of people (Khaniabadi et al. 2017). Recent studies have highlighted that one of the most dangerous components of air pollution to human health is particulate matter (PM), a mixture of particles that are suspended in the air (Bell et al. 2011). These pollutants can be classified according to the diameters of the particles: large PM (10–100 μm), coarse PM (2.5–10 μm), fine PM (0.1–2.5 μm), and ultra-fine PM (< 0.1 μm) (Power et al. 2009; Popek et al. 2013). PM is formed by both natural forces (volcanic activity, forest fires, etc.) and human activity; however, PM of human origin is frequently enriched by heavy metals, polycyclic aromatic hydrocarbons and other pollutants (Jouraeva et al. 2002; Alghamdi 2016). Industrial areas, urban agglomerations and high-traffic areas are characterised by the highest degree of PM contamination (Janssen et al. 1997; Popek et al. 2015).

Beyond technical equipment such as chimney and car particle filters, plants, especially deciduous trees and shrubs, have an important role in reducing the amount of PM in the atmosphere by accumulating pollutants on the surface of their leaves (Popek et al. 2017a). Their crowns also inhibit and block the spread of PM in the air. Numerous studies have shown that plants are efficient passive PM collectors (Sæbø et al. 2012; Popek et al. 2013, 2015; Przybysz et al. 2014), but species differ in their ability to accumulate PM (Dzierżanowski et al. 2011; Sæbø et al. 2012; Popek et al. 2013; Song et al. 2015). Deposition of PM depends mostly on the density of the plant (quantity and size of leaves) and the morphological structure of the leaf surface (Prusty et al. 2005). It can also be increased by the presence of epicuticular waxes, in which PM can become stuck or immersed (Dzierżanowski et al. 2011; Leonard et al. 2016). The presence of PM on leaves has an adverse effect on plants, mainly by limiting the amount of light reaching the mesophyll, which is reflected in a decline in the efficiency of photosynthesis (Przybysz et al. 2014; Saadullah et al. 2014; Popek et al. 2017b). Moreover, PM may clog the respiratory tract and even penetrate the plant tissues (Burkhardt and Grantz 2016). Large amounts of accumulated particles affect the physiological processes of the entire plant, as well as organisms on upper trophic levels, such as folivorous insects (Khan et al. 2013). Surprisingly, this aspect remains underdeveloped. It is difficult to estimate the effects of air pollutants on herbivores in nature because such organisms are exposed to a wide range of uncontrolled factors (e.g., weather conditions, parasitoids, mixtures of pollutants); it is therefore important to investigate the effects of air pollutants under experimental conditions (see future directions in Talley et al. 2006).

The effects of PM and heavy metal contamination on herbivorous insects are often different and depend on the insect guild involved. It is assumed that cambio- and xylophages achieve better growth and faster development on plants stressed by particle accumulation (indirect effect of PM via influence on host). The opposite relationship is usually observed in folivorous and gall-inducing insects (effect of indirect and direct influence of PM; Khan et al. 2013). PM is lethal for folivores, or at least causes starvation or desiccation of the insects (Flanders 1941). The most common negative effects for folivores are increased mortality, body mass loss and decreased fertility (Khan et al. 2013). Increased PM accumulation on leaves leads to clogs in the digestive system and a decrease in the use of resources necessary to grow and develop an insect (Flanders 1941). Additionally, insects must increase their production of metabolites involved in the biochemical immune response (van Ooik et al. 2007). Several studies, however, have reported that outbreaks of herbivores are more frequent on polluted hosts, such as roadside trees or shrubs (Pringle et al. 2014). This is likely because there is a higher nitrogen concentration under such conditions, which improves food quality for insect herbivores (Port and Thompson 1980). Another reason may be the preference of some insect species for the high-sunshine conditions found on trees along the road. Under such conditions, insect performance is better (Łukowski et al. 2014, 2015a).

The genus *Prunus* L. plays a key role in the species composition of the understory in European forests (Houston and Caudullo 2016; Aerts et al. 2017). Particularly widespread are shrubs of *P. padus* L. (bird cherry) and the closely related *P. serotina* Ehrh. (black cherry). The former is a species native to Europe, with a wide geographic range (Łukowski et al. 2014; Houston and Caudullo 2016), whereas black cherry is an alien and very invasive species in Europe, originating from the north-eastern and central parts of the USA, Mexico and north of South America (Pairon et al. 2010). Shrubs of both *Prunus* species are heavily damaged by many folivores (Leather 1996; Uusitalo 2004; Meijer et al. 2012), especially the leaf beetle *Gonioctena quinquepunctata* (Coleoptera: Chrysomelidae) (Halarewicz and Jackowski 2011; Mąderek et al. 2015; Schilthuizen et al. 2016) and aphids *Rhopalosiphum padi* (Heteroptera: Aphididae) (Halarewicz and Gabryś 2012). The monophagous ermine moth *Yponomeuta evonymellus* L. (Lepidoptera: Yponomeutidae) attacks mainly *P. padus* (Leather 1986; Łukowski et al. 2014), but in recent years has also been found on *P. serotina* (Karolewski et al. 2014, 2017). The leaf beetle and both plant species make good model systems with which to study the impact of PM pollution, as well as the interaction of native and non-native plant species from the perspective of accumulation and the response of herbivores to PM pollution. Their leaves are known to attract PM present in the air and can curtail atmospheric air pollution, especially in urban environments (Popek et al. 2017b). Differences in the morphology of both species’ leaves, however, may potentially explain the higher accumulation of PM on the foliage of *P. padus* (Danielewicz and Wiatrowska 2013; Popek et al. 2017b). Both plants are also found in similar natural habitats in Poland (Dyderski and Jagodziński 2015; Łukowski et al. 2017); both are very common in the understory of urban forests and urban plantings (Dyderski et al. 2016), and often choose well-lit forest edges or roadsides (Mizera et al. 2016). Moreover, the leaves of *P. padus* and *P. serotina* are damaged by *G. quinquepunctata* more so than those of shrubs of other understory species (Karolewski et al. 2013).

The aim of this study was (i) to assess the response of the polyphagous leaf beetle *G. quinquepunctata* to the change of food quality caused by dusting the leaves of native bird cherry (*P. padus*) and non-native black cherry (*P. serotina*). Leaves were experimentally polluted with PM from different sources: cement plant or roadside. Additional research objectives were (ii) to assess total accumulation of PM on foliage of both species and (iii) to investigate any change in the chemical composition of leaves after experimental pollution. We hypothesised that (1) PM has a negative impact on the growth and development of *G. quinquepunctata*, and the effect is greater on *P. padus* than on *P. serotina*; (2) PM accumulation on leaves is higher on *P. padus* than that on *P. serotina*; and (3) the composition of the main attractants and repellents in leaves significantly differs after treatment with PM.

## Materials and methods

### Plant material and study area

Studies were conducted in 2016 on *P. padus* L. and *P. serotina* Ehrh. We used 9-year-old seedlings of both *Prunus* species of local origin (seeds from the Palędzie Forest 52°23′N, 16°40′E, sown in 2007). Every seedling chosen for the experiment was similar in size, had an average height of 50 cm, was planted in a pot (15 dm^3^) and was in good condition (healthy and free from pests).

To protect plants from outside stress factors, and especially from accumulation of particulate matter (PM) from the air, they were placed in a greenhouse (18 m^2^) with appropriate growing conditions. Plants were divided into three groups, each with ten individuals of each species (Σ *n* = 60). Seedlings in the first two groups were dusted in early May with an equal volume (50 cm^3^) of PM, with a diameter of less than 100 μm (prior to treatment, the PM was sieved through 100 μm), which was collected from the roadside (ROAD variant) and from a cement plant (CEMENT variant). The roadside PM was collected from the edge of a busy road (Matyi and Bolesława Krzywoustego) in Poznań, Poland (52°24′N, 16°56′E), whereas the cement PM consisted of sieved Portland cement particles (Cemmas A.S., Poland). The third group consisted of control plants (CONTROL variant) not subjected to any stress conditions. All seedlings were watered as necessary throughout the growing season, only by supplying water to the soil.

### Analysis of PM accumulation on leaves

In order to estimate the PM accumulation on the leaves of both species cultivated in different variants, we determined the total amount of PM, defined as the sum of two categories of PM on leaves: washable with water–surface PM (_S_PM), and washable with chloroform–in-wax PM (_W_PM). For each variant in mid-May, 1 week after dusting (immediately before an experiment with insects), a few leaves were harvested from seedlings (Σ *n* = 3 PM source variants × 2 *Prunus* species × 10 seedlings). In order to obtain sufficient material, the leaf area per sample ranged from 300 to 400 cm^2^. Samples consisted of leaves gathered from different parts of the canopy to be representative of the seedlings. Leaves were placed in paper bags, labelled and kept at ambient temperature until analysis. Each leaf sample was washed with water and then with chloroform. Both categories (_S_PM and _W_PM) were measured in 0.2–100 μm size fraction. The liquids were filtrated using three types of paper filters: Type 91 and Type 42 paper filters and PTFE membrane filters (all Whatman, UK), with pore sizes of 10, 2.5 and 0.2 μm, respectively, to avoid filter blockage. Before and after filtration, all filters were dried and weighed. For further calculations, we took the sum of the mass of PM from the three filters of different fractions. Leaves were scanned and their areas were measured in WinFOLIA 2004 (Regent Instruments Inc., Canada). The amounts of PM were then expressed in milligrammes per square centimetre.

### Insects and laboratory experiment

This study was conducted on *G. quinquepunctata* Fabricius (syn. *Phytodecta quinquepunctata* Kirby [Coleoptera: Chrysomelidae] (Urban 1998)). We used all larval stages, pupae and adult beetles in this laboratory experiment. Larval *G. quinquepunctata* were selected randomly in the field near Poznan, in Zalasewo (Kobylepole Forest, Babki Forest District; 52°36′N, 17°06′E) at the earliest possible stage of development (mid-May), and their initial mass was recorded. The larvae were collected from the respective species (Σ *n* = 30 individuals × 2 host plant species), due to the broadly reported influence of the first food consumed by larvae on their performance in later stages of development (Fortuna et al. 2013). The larvae were then kept singly in Petri dishes, reared at room temperature and fed leaves of the given variant (species and different source of PM pollution) from the beginning of the experiment (May, 17) until the day of their death or the beginning of the winter diapause. The petiole of each leaf was placed in an Eppendorf tube filled with water, through a hole in its lid. The leaf was replaced with a new one every 2 days.

To determine insect performance and growth indicators, we used various parameters of growth and development (Waldbauer 1968; Scriber and Slansky 1981). Every day, data concerning developmental stages and larval mortality were recorded to assess the duration of development (DD)—that is, the time from the beginning of the experiment to pupation—and the pupal period, the time from pupation to the emergence of adult beetles. Every 2 days, larval, pupal and adult masses were measured with an analytical balance (± 0.01 mg, CP225D; Sartorius, Göttingen, Germany). In the “Results” section, we compared the maximum values of larval, pupal and adult masses recorded during the experiment.

Total food eaten (TFE; in g dry mass of leaves) is the difference between the estimated dry mass of the leaf before it was placed in an Eppendorf tube and the dry remains. For the estimation, the fresh weight of a leaf was compared to its dry weight after desiccation at 65 °C, to calculate the fresh/dry weight ratio (for each variant and date). TFE was calculated by summing the estimated mass of food eaten by the larvae from the beginning of the experiment to pupation. Consumption index (CI) was based on the formula CI = TFE/DD × *A*, where *A* is the fresh mass of larvae during the feeding period. Relative growth rate (RGR) was calculated using the following formula: RGR = (*M*_t_ – *M*_0_)/(DD × *A*), where *M*_0_ and *M*_t_ denote initial and final larval mass, respectively (in mg). Based on the maximal larval mass and TFE, we also defined the efficiency of conversion of ingested food (ECI), using the following formula: ECI = (larval mass/TFE) × 100%. More details on the methods and parameters that we used in this study can be found in our earlier reports (Łukowski et al. 2015a; Mąderek et al. 2015) and in the literature cited therein.

### Chemical analysis of food quality

The following chemical components of leaves were determined: nitrogen concentration (N); total non-structural carbohydrates (TNC), as a sum of soluble sugars and starch (ST); soluble phenolic compounds (TPh) and condensed tannins (CT). The material was collected on two dates: 1 week after dusting the seedlings (in mid-May) and at the end of the experiment (in mid-July), from the same individuals from which leaves were collected for laboratory experiments.

The analyses utilised powdered leaf tissue, obtained from leaves previously dried at either 40 °C for the condensed tannins, or at 65 °C for the other compounds. The concentration of total soluble phenols was measured colourimetrically using Folin and Ciocalteu’s Phenol Reagent (SIGMA F-9252), following the method of Johnson and Schaal (1957) modified by Singleton and Rossi (1965). Condensed (catechol) tannins were measured using a colour reaction with vanillin in an acid medium (Price et al. 1978). Results of the phenol measurements were expressed per micromolar of chlorogenic acid in per gramme of dry mass (d.m.), whereas condensed tannins were converted into micromolar of catechin per gramme d.m. Nitrogen (N) content (% d.m.) was determined using an Elemental Combustion System CHNS-O 4010 analyser (Costech Instruments, Pioltello, Italy). Total non-structural carbohydrates (soluble carbohydrates and ST) were determined as described by Haissig and Dickson (1979) and Hansen and Møller (1975). Soluble carbohydrates were assayed in methanol-chloroform-water extracts and TNC results were expressed as % d.m., or for ST as ‰ d.m. Absorbances (tannins, phenols and carbohydrates) were determined with a spectrophotometer (UV-1700 Visible Spectrophotometer; PharmaSpec, Shimadzu, Japan). Detailed descriptions of the methods for these chemical analyses are described in our previous paper (Karolewski et al. 2013).

### Statistical analysis

A two-way ANOVA model was used to compare the amount of PM from leaves of saplings (both *Prunus* species) treated with PM from different sources. Three-way ANOVA with mixed effects was used to compare the content of nitrogen, condensed tannins, soluble phenols and non-structural carbohydrates in leaves from saplings (both *Prunus* species) treated with PM from different sources, on two dates (fixed effects), and saplings were considered a random effect.

Survival analysis (Log-Rank test) was used to determine the probability of survival of larvae over time. A four-way ANCOVA model was used to compare the larval, pupal and adult masses, DD, pupal period, TFE, CI, RGR and ECI of insects of both sexes, fed on leaves from different *Prunus* species treated with PM from different sources. The initial larval mass was used as a covariate. Data expressed as percentages were transformed to meet linear model assumptions using a formula proposed by Bliss (1938). All calculations were performed using JMP Pro 13.0 software (SAS Institute Inc., Cary, NC, USA).

## Results

### PM accumulation on leaves

**Fig. 1:**
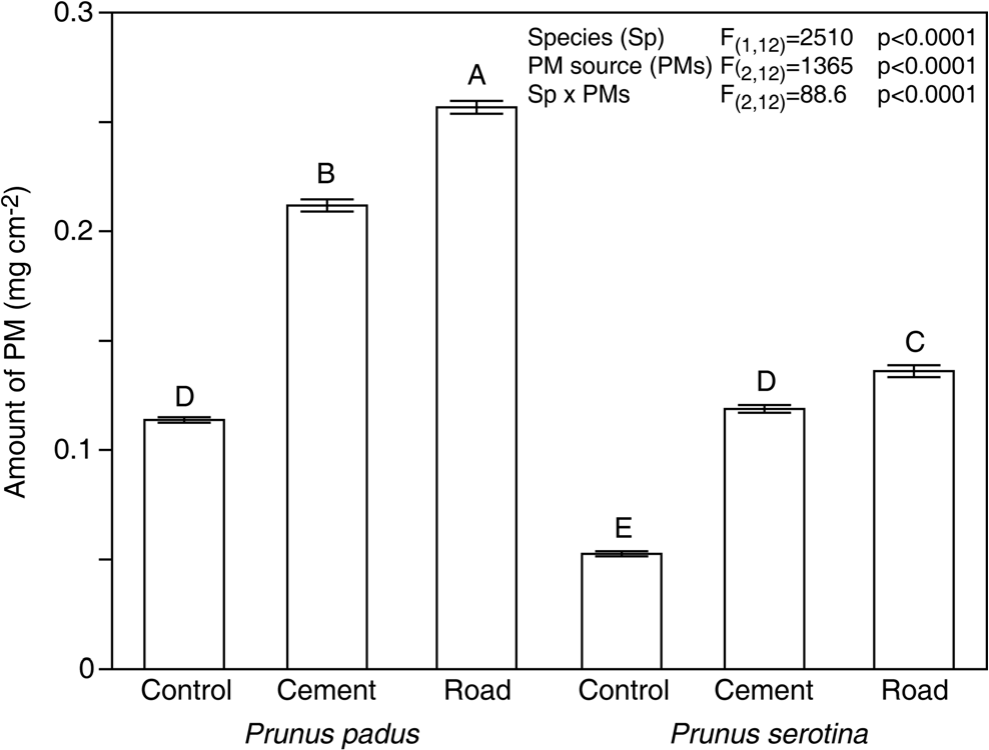
Mean, standard error and ANOVA results for total particulate matter (PM; sum of surface and in-wax PM, in the initial term) on leaves of *Prunus padus* and *P. serotina* seedlings growing under stress from different sources of PM pollution. Levels not connected by the same letter are significantly different (Tukey’s HSD test)

For both tested plant species, PM from two categories (surface PM, _S_PM, and in-wax PM, _W_PM) was found. We found that regardless of species, a greater amount of PM was accumulated on the road and cement variants, and less on the controls. The results also documented differences in PM accumulation between leaves of *P. padus* and *P. serotina* (Fig. 1). The former accumulated approximately 116% more PM than the control, and 82% more cement PM and 94% more road PM than the latter. Even the control plants, although shielded from external contamination, accumulated a certain amount of PM. In both species, PM was found both as surface PM (_S_PM) and as in-wax PM (_W_PM). Species had a significant effect on the amount of _S_PM (F_(1, 28)_ = 129.4; *p* < 0.0001) and _W_PM (F_(1, 28)_ = 14.1; *p* < 0.0001) present on leaves. In both *Prunus* species, more PM was deposited on the leaf surface than was immobilised in waxes. The ratio of _S_PM to _W_PM was 2.1:1 in *P. padus* and 1.6:1 in *P. serotina*, regardless of the origin of PM.

### Chemical analysis of food quality

For the laboratory experiment, we determined whether the food quality (nutritive value) of leaves of *Prunus* seedlings is influenced by PM pollution, and thus whether it can have an effect on larval growth and development. We showed little influence of PM on the quality of food. We found marginally significant differences in the concentration of nitrogen (N) and total non-structural carbohydrates (TNC) between the PM source variants (Table 1). A little more TNC was stored in the leaves of the cement variant, and there was no difference between the control and road variants. In addition, we observed that *P. padus* leaves in the cement dust variant accumulated more TNC than in the other variants, whereas in *P. serotina*, PM source variant had no significant effect on this group of primary metabolites. The concentration of starch (and soluble sugars) showed the same relationship as that of TNC.

**Table 1:** Mean values (±SE) of nitrogen (N), condensed tannins (CT), total phenols (TPh) content, and total non-structural carbohydrates (TNC) and starch (ST) concentration in the leaves of two different *Prunus* species, growing under stress from different sources of particulate matter pollution, at two dates of collection (see “Materials and methods”), as well as a summary of ANOVA results

PM source variant			N (%)		CT (μM g^−1^ d.m.)		TPh (μM g^−1^ d.m.)		TNC (%)		ST (‰)	
			*Prunus padus*	*Prunus serotina*	*Prunus padus*	*Prunus serotina*	*Prunus padus*	*Prunus serotina*	*Prunus padus*	*Prunus serotina*	*Prunus padus*	*Prunus serotina*
Control			3.61 (0.17)	3.69 (0.31)	41.83 (4.58)	86.83 (32.25)	164.9 (14.9)	194.8 (24.6)	7.37 (0.36)	8.15 (0.50)	4.525 (0.036)	4.579 (0.062)
Road			3.57 (0.33)	3.52 (0.32)	37.36 (6.18)	90.70 (40.24)	178.6 (10.7)	203.8 (16.9)	7.22 (0.26)	8.36 (0.35)	4.556 (0.038)	4.644 (0.055)
Cement			3.67 (0.32)	3.67 (0.28)	41.93 (5.19)	49.39 (6.60)	170.2 (10.7)	183.4 (6.7)	8.99 (0.34)	8.23 (0.53)	4.508 (0.036)	4.611 (0.067)
ANOVA	df	df error	*F*	*p*	*F*	*p*	*F*	*p*	*F*	*p*	*F*	*p*
Species (Sp)	1	12	0.081	0.780	6.610	*0.025*	7.156	*0.020*	1.427	0.255	5.979	*0.031*
PM source (PMs)	2	12	3.286	0.073	0.811	0.468	1.059	0.377	2.916	0.093	0.795	0.474
Sp × PMs	2	12	0.805	0.470	1.058	0.378	0.337	0.720	3.208	0.077	0.188	0.831
Date (D)	1	12	215.2	*< 0.001*	8.622	*0.013*	4.424	0.057	11.21	*0.006*	17.44	*0.001*
Sp × D	1	12	0.512	0.488	14.44	*0.003*	29.21	*< 0.001*	25.46	*< 0.001*	9.227	*0.010*
PMs × D	2	12	2.083	0.167	1.743	0.217	6.071	*0.015*	0.503	0.617	3.347	0.070
Sp × PMs × D	2	12	1.704	0.223	1.583	0.245	4.860	*0.028*	0.267	0.770	0.250	0.783

p values < 0.05 are in italics

We found a significant influence of *Prunus* species on the concentrations of substances that were favourable for insect development (starch), and of plant defence compounds (tannin and phenol concentrations). During the grazing period, leaves of *P. serotina* had 2% more starch than did those of *P. padus* (Table 1). *P. serotina* also had 87% higher concentrations of condensed tannins (CT) and 13% higher concentrations of soluble phenolic compounds (TPh) than did *P. padus*. We did not observe significant effects of host species on the N and TNC content of leaves; however, in the case of TNC, there was a marginally significant interaction of species × PM source.

As expected, the date of collection of the leaves had a significant influence on several of the studied metabolites (Table 1). We found an increase in tannin concentration over time; tannin levels were 49% higher at the end of the experiment (in mid-July) than 1 week after dusting the seedlings (in mid-May). In addition, we discovered a decrease over time in the concentration of nitrogen, TNC and starch: decreases of 29, 8 and 3%, respectively.

### Laboratory experiment

**Fig. 2:**
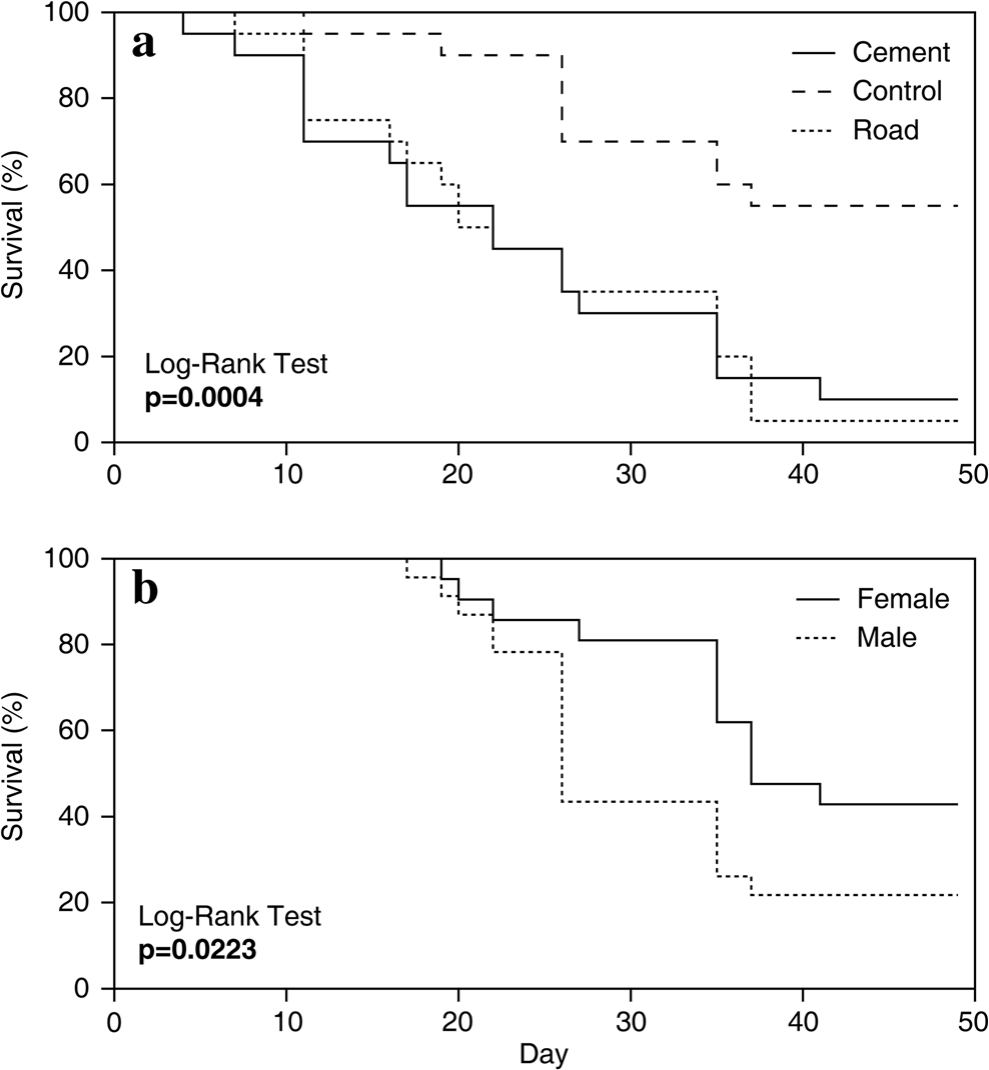
The survival analysis (Log-Rank test) of *Gonioctena quinquepunctata* **a** larvae and adults feeding on leaves of seedlings (average of two *Prunus* species), growing under stress from different sources of particulate matter (PM) pollution, and **b** only adults of both sexes (average of all variants of PM source). *p* values < 0.05 are in bold

Approximately 60% of insects not exposed to PM survived. We found that PM pollution affected insect survival, as only 10% of insects exposed to PM survived (Fig. 2a). We also noted that sex impacted survival‐40% of females and 20% of males survived (100% means only those insects that pupated; Fig. 2b). We found no significant impact of *Prunus* species on survival of insects (Log-Rank test; *p* = 0.99).

We found that neither *Prunus* species nor PM source variant significantly affected masses of larvae and pupae, duration of larval and pupal development or relative growth rate (RGR; Table 2). Significant influences of the aforementioned factors were noted for adult insect masses and the efficiency of conversion of ingested food (ECI), however. Adult insect mass was 3% higher on *P. serotina* leaves than that on *P. padus*. Additionally, the masses of adult insects on control leaves were 16 and 28% higher than those on cement and road PM variants, respectively. Moreover, in the case of adult mass, there was a significant interaction of species × PM source. The ECI was 4% higher on *P. padus* leaves than that on *P. serotina*. In addition, ECIs on control leaves were 24 and 51% higher than those on the cement and road PM variants, respectively. We observed that the abovementioned relationships (i.e. higher ECI on control leaves) corresponded well with total food eaten (TFE) and consumption index (CI) in the case of the PM source variant. In the road and cement variants, for which ECI were lower, larvae compensated for this by eating a greater total amount of food. The TFEs were 28 and 41%, and CIs were 29 and 41%, lower on control leaves, compared to the cement and road PM variants, respectively.

**Table 2:** Mean values (±SE) of maximum larval, pupal and adult mass, duration of larval development (DD), pupal period, total food eaten (TFE), consumption index (CI), efficiency of conversion of ingested food (ECI) and relative growth rate (RGR) of *Gonioctena quinquepunctata* feeding on leaves of *Prunus padus* or *P. serotina*, growing under stress from different sources of particulate matter pollution, as well as a summary of ANCOVA results. Initial larval mass (ILM) was used as a covariate to adjust for initial mass differences among insects. Levels not connected by the same letter are significantly different (Tukey’s HSD test for effect “Sp × PMs”). *p* values < 0.05 are in italics

PM source variant		Larval mass (mg)		DD (day)		Pupal mass (mg)		Pupal period (day)		Adult mass (mg)		TFE (mg dry mass)		CI (g g^−1^ day ^−1^)		ECI (g g^−1^100%)		RGR (g g^−1^ day ^−1^)	
		*Prunus padus*	*Prunus serotina*	*Prunus padus*	*Prunus serotina*	*Prunus padus*	*Prunus serotina*	*Prunus padus*	*Prunus serotina*	*Prunus padus*	*Prunus serotina*	*Prunus padus*	*Prunus serotina*	*Prunus padus*	*Prunus serotina*	*Prunus padus*	*Prunus serotina*	*Prunus padus*	*Prunus serotina*
Control		15.14 (0.65)	14.51 (1.00)	10.2ab (0.7)	11.6a (0.4)	12.80 (0.75)	12.27 (0.68)	5.9a (0.4)	5.0a (0.4)	16.45a (1.26)	15.13a (0.80)	28.78 (1.03)	26.99 (1.45)	0.17b (0.02)	0.20b (0.01)	51.34 (1.17)	51.65 (1.72)	0.012 (0.009)	0.010 (0.009)
Road		15.57 (0.60)	14.45 (0.70)	11.0a (0.7)	10.0ab (0.8)	12.44 (0.64)	12.98 (0.61)	4.4b (0.2)	5.8a (0.6)	11.22d (0.98)	13.02bc (0.45)	47.72 (4.05)	47.00 (5.43)	0.33a (0.05)	0.30ab (0.05)	34.37 (3.18)	33.87 (2.34)	0.015 (0.008)	0.027 (0.004)
Cement		15.09 (0.60)	14.89 (1.18)	9.8b (0.7)	10.0ab (0.8)	12.38 (0.95)	13.87 (0.91)	7.0a (0.1)	6.0a (0.7)	12.08cd (1.04)	14.60b (0.45)	35.52 (1.19)	41.81 (4.70)	0.28ab (0.02)	0.25ab (0.04)	43.75 (1.04)	39.09 (2.36)	0.006 (0.004)	0.009 (0.008)
ANCOVA	df	*F*	*p*	*F*	*p*	*F*	*p*	*F*	*p*	*F*	*p*	*F*	*p*	*F*	*p*	*F*	*p*	*F*	*p*
Species (Sp)	1	0.007	0.936	0.021	0.885	0.006	0.938	1.138	0.295	6.392	*0.017*	3.307	0.079	3.012	0.093	7.679	*0.010*	0.081	0.778
PM source (PMs)	2	0.631	0.539	1.514	0.236	0.130	0.878	2.663	0.086	45.21	*< 0.001*	12.33	*< 0.001*	6.091	*0.006*	24.88	*< 0.001*	1.828	0.178
Sex (S)	1	6.906	*0.013*	1.086	0.306	11.23	*0.002*	0.073	0.789	63.18	*< 0.001*	3.727	0.063	0.005	0.943	0.840	0.367	6.064	*0.020*
Sp × PMs	2	0.252	0.779	6.179	*0.006*	0.111	0.895	7.232	*0.003*	4.749	*0.016*	1.009	0.377	3.396	*0.047*	1.188	0.319	0.185	0.832
Sp × S	1	0.010	0.920	0.120	0.732	0.010	0.921	0.023	0.880	3.066	0.090	0.001	0.977	0.160	0.693	0.014	0.907	0.088	0.769
PMs × S	2	0.001	0.999	0.067	0.935	0.695	0.507	2.116	0.138	7.942	*0.002*	0.701	0.504	0.252	0.779	0.743	0.484	0.042	0.959
Sp × PMs × S	2	0.198	0.821	0.225	0.800	0.038	0.963	0.100	0.905	0.066	0.937	0.208	0.813	0.227	0.798	1.363	0.271	0.010	0.990
ILM	1	2.711	0.110	26.60	*< 0.001*	3.536	0.070	14.37	*< 0.001*	0.465	0.500	4.321	*0.046*	9.882	*0.004*	1.059	0.311	101.9	*< 0.001*
df error		31		31		31		30		31		30		30		30		30	

In all three developmental stages, sex had a significant effect on insect mass. Female mass was greater in all treatments, but differences in adult mass between females and males were not the same among the variants (there was a significant interaction of PM source × sex). For the control variant, the differences between female and male masses were larger than those for both variants with PM treatments.

## Discussion

These results show that PM deposited on the leaves of both *Prunus* species, and consumed by *G. quinquepunctata*, has a significant impact on the insects’ development, including a higher mortality rate; however, the effect was most evident in the adult stage. It was found that *Prunus* species and PM pollution did not affect the duration of development (DD) and pupal period, or the body mass of larvae and pupae. *Prunus* species and PM source variant did, however, significantly affect adult insect masses and the efficiency of conversion of ingested food (ECI), which corresponded well with the total food eaten (TFE) and consumption index, in the case of PM source variant. The results clearly demonstrate the harmful impact of PM accumulated by *Prunus* leaves on the mortality and growth of their main herbivorous species, *G. quinquepunctata*. Differences in the level of PM accumulation by these plant species, however, do not significantly affect this insect.

We found two types of PM accumulation: that on the leaf surface and an in-wax layer. Even control plants, although protected from external contamination, accumulated a certain amount of PM, likely from air exchange between the greenhouse and the outside. In this study, we confirmed our second hypothesis that leaves of *P. padus* accumulated more PM than *P. serotina*. These results are consistent with our previous observations of the PM accumulation pattern on *Prunus* leaves (Popek et al. 2017b). We think that the differences in the studied species leaf anatomy and the morphology are the main reasons for greater accumulation by *P. padus* (e.g. *P. padus* leaves are distinctly wrinkled, with numerous hollows and ridges, in contrast to the smooth and slippery leaves of *P. serotina*; Danielewicz and Wiatrowska 2013). Prusty et al. (2005) also observed significant variations in PM interception ability among plant species and concluded that the interception capacity of plants depends mainly on their canopy shape and size, as well as leaf surface characteristics. Bakker et al. (1999) reported that *Plantago* species with smooth leaves accumulated smaller amounts of PM than did species with rough leaves. Thus, differences in the structure of both *Prunus* species’ leaves could explain the differences in PM accumulation on the foliage of native *P. padus* and alien *P. serotina*.

All air pollutants directly affect plants, mainly through leaves, but also indirectly through soil (Steubing et al. 1989). PM causes both chemical and physical effects on plants, and the chemical effects are greater when the PM contains many different toxins and heavy metals from roadside and industrial emissions (Przybysz et al. 2014; Popek et al. 2017a). Accumulated PM affects the physiological processes of the entire plant, primarily by limiting light access to the leaves and clogging the stomata (Su and Sun 2006; Squires 2016). Thus, dust-covered plants are expected to display symptoms such as transpiration inhibition, reduced photosynthesis, increased water loss and reduced vegetative and reproductive growth. In our previous study, the rates of photosynthesis and stomatal conductance were negatively affected by both road and cement PM in *P. padus*, but in *P. serotina* no negative impact was found (Popek et al. 2017b). Reduction in the concentration of chlorophyll, which directly affects general plant productivity (Saadullah et al. 2014), shows protein, total carbohydrate, starch and phytomass reduction (Prasad and Inamdar 1990; Raajasubramanian et al. 2011). These stress symptoms could influence herbivores through mortality of host plants or through a change in host plant quality (Khan et al. 2013). The current results do not, however, show significant differences in the content of the studied elements and metabolites, such as nitrogen (N), phenols, tannins and starch (ST; Table 1). Slightly more N and total non-structural carbohydrates (TNC) were stored in the leaves of the cement dust variant, which is contrary to observations made by most previous authors (Prasad and Inamdar 1990; Raajasubramanian et al. 2011; Salama et al. 2011). The higher accumulation of TNC in *P. padus* cement variant leaves is likely associated with impaired reducing sugar export from the mesophyll, as was observed in *Datura inoxia* Mill. growing under cement factory emissions (Salama et al. 2011). Overall, we negatively verified our third hypothesis because the composition of commonly studied metabolites responsible for the quality of food for herbivores did not significantly differ after experimental pollution. This does not exclude the possibility that a longer period of accumulation of PM could cause differences in the content of these metabolites in the leaves of both *Prunus* species to occur.

It is a known fact that the leaves of both studied plant species have comparable nutritional value (similar TNC, ST and N concentration); however, levels of defensive compounds, such as phenols and condensed tannins, are higher in the alien *P. serotina* than those in the native *P. padus* (Karolewski et al. 2013; Mąderek et al. 2015). Collectively, the results of the present study are consistent with those of previous studies connected with food quality, showing that experimental PM pollution does not alter the concentrations of the studied defence metabolites in either species. Moreover, *G. quinquepunctata* is able to tolerate higher levels of defence compounds in *P. serotina*, as evidenced by the results of our current and previous laboratory experiments involving the breeding of larvae and adult insects on both *Prunus* species (Mąderek et al. 2015).

Sex had an important influence on the insect mass of all studied stages of development, as well as on relative growth rate (RGR; Table 2). In general, the females of most insect species are much heavier than their male counterparts (Blanckenhorn 2000, 2005), and this has also been determined to be true for female beetles in earlier studies of *G. quinquepunctata* (Łukowski et al. 2015b).

Contrary to our expectations in our laboratory experiment, neither PM pollution nor host species significantly affected DD, pupal period, RGR or larval or pupal masses of *G. quinquepunctata*. This lack of effect of dust on the growth and development of larvae and pupae may be due to the relatively short period of the larval stage and the inert response of pupae at these stages (average 10 and 5 days, respectively), compared to adult life (on average over 21 days). We found that PM pollution significantly decreases the mass of adult insects. Lower insect mass is the most frequently used indicator of worse feeding conditions (Lee and Roh 2010; Kaplan et al. 2014). We can certainly say that PM-polluted leaves provide a poorer quality source of food for *G. quinquepunctata*, due to their obligatory consumption of leaf tissue with accumulated PM, which resulted in decreased body mass and survival. In several previous reports, insects fed with leaves with accumulated cement or roadside dust reached lower body masses and their survival rate decreased (Zvereva and Kozlov 2010; Khan et al. 2013). There are many probable causes for the lower mass gained and the decreased survival of insects, but mainly the negative effects of heavy metal have been reported (Boyd and Martens 1994; Coleman et al. 2005; van Ooik et al. 2007). Additionally, previous researchers mention other reasons such as mechanical clogging of the digestive system (Flanders 1941), increased production of metabolites involved in the biochemical immune response (van Ooik et al. 2007), the desiccation of the insect (Flanders 1941; Ebeling 1971) and mechanical hindrance of movement by the particle coating (Negri et al. 2015). Moreover, lower food quality also leads to a lower ECI parameter (Giertych et al. 2005; Tremmel and Müller 2013). In this study, *G. quinquepunctata* larvae fed with PM-polluted leaves compensated for their lower ECI by increasing their TFE. This is consistent with the assumption that insects that feed on lower quality leaves compensate by consuming more food (Woods and Kingsolver 1999). In our previous studies, the lower ECI value of *G. quinquepunctata* larvae feeding on sunlit leaves of both *Prunus* species (lower quality) was compensated for by their higher level of consumption (Mąderek et al. 2015). Body mass are correlated with size and both strongly depend on food quality (Thomas et al. 1980). In this study, we found a higher mass of insects on *P. serotina.* Also, Meijer (2013) found the bigger adult size on *P. serotina*, although the comparison there was with *Sorbus* L., not with *P. padus*. Morphological plasticity, as it is affected by host quality and environment parameters, may be important for the movement and survival of an insect population (Taylor and Merriam 1995).

In general, lower nutritive value leaves usually result in an extended duration of larval development (Khan et al. 2013; Tremmel and Müller 2013), which increases insect vulnerability due to attack by predators and parasites, according to the “slow-growth high-mortality” hypothesis (Chen and Chen 2016). In our research, DD was not extended significantly enough to change the time of exposure to parasites. We therefore believe that in nature, it is also possible that the dusting of leaves would not increase the exposure of larvae to parasites. It has been suggested that elevated PM concentrations in the air significantly affect parasitoids, especially from the order Hymenoptera (Alstad et al. 1982). It is believed that in this specific environment, pathogens, such as viruses, play a role in the natural control of herbivore populations (Olofsson 1988; Khan et al. 2013). Overall, we partly positively verified our first hypothesis because PM pollution has a negative impact on survival rate at all stages of development, as well as on the growth of adult insects, but not on the growth and development of larvae and pupae. We fully positively confirmed the second part of this hypothesis, however, because the effect was greater in *P. padus* than in *P. serotina*.

In conclusion, it is important to note that PM pollution significantly affects the survival and the mass of adult *G. quinquepunctata*. The lower mass of adult insects feeding on dusted leaves of *P. padus* demonstrates that this species provides poorer conditions for individuals of *G. quinquepunctata* under PM pollution stress than does the alien *P. serotina*. Our study also revealed that leaves of native *P. padus* have a better ability to accumulate pollutants in comparison with those of non-native *P. serotina*, and therefore could potentially enhance the quality of an urbanised environment. These results are consistent with the generally accepted view that the vegetation in cities plays an important role in cleaning the atmosphere (Nowak et al. 2006). The optimal utilisation of trees and other plants to purify the environment should therefore be a priority for architects and urban planners, and it is worth considering planting *P. padus*, as it is a native species. In Europe, *P. serotina* is classified as a highly invasive species causing significant transformation of ecosystems’ species composition, as well as difficulties in forest management (Vanhellemont et al. 2009). Moreover, given the important role of insects in the transfer of energy from plants to higher trophic levels, it is desirable to investigate the influence of contamination on insect biomass.
